# *Suaeda vermiculata* Aqueous-Ethanolic Extract-Based Mitigation of CCl_4_-Induced Hepatotoxicity in Rats, and HepG-2 and HepG-2/ADR Cell-Lines-Based Cytotoxicity Evaluations

**DOI:** 10.3390/plants9101291

**Published:** 2020-09-29

**Authors:** Salman A. A. Mohammed, Riaz A. Khan, Mahmoud Z. El-Readi, Abdul-Hamid Emwas, Salim Sioud, Benjamin G. Poulson, Mariusz Jaremko, Hussein M. Eldeeb, Mohsen S. Al-Omar, Hamdoon A. Mohammed

**Affiliations:** 1 Department of Pharmacology and Toxicology, College of Pharmacy, Qassim University, Qassim 51452, Saudi Arabia; hu.ali@qu.edu.sa; 2 Department of Medicinal Chemistry and Pharmacognosy, College of Pharmacy, Qassim University, Qassim 51452, Saudi Arabia; m.omar@qu.edu.sa; 3 Department of Clinical Biochemistry, Faculty of Medicine, Umm Al-Qura University, Makkah 21955, Saudi Arabia; mzreadi@uqu.edu.sa; 4 Department of Biochemistry, Faculty of Pharmacy, Al-Azhar University, Assiut 71524, Egypt; 5 King Abdullah University of Science and Technology (KAUST), Core Labs, Thuwal 23955-6900, Saudi Arabia; abdelhamid.emwas@kaust.edu.sa (A.-H.E.); salim.sioud@kaust.edu.sa (S.S.); 6 King Abdullah University of Science and Technology (KAUST), Biological and Environmental Sciences and Engineering Division (BESE), Thuwal, 23955-6900, Saudi Arabia; benjamingabriel.poulson@kaust.edu.sa (B.G.P); mariusz.jaremko@kaust.edu.sa (M.J.); 7 Department of Biochemistry, Faculty of Medicine, Al-Azhar University, Assiut, 71524, Egypt; 8 Medicinal Chemistry and Pharmacognosy Department, Faculty of Pharmacy, JUST, Irbid 22110, Jordan; 9 Department of Pharmacognosy, Faculty of Pharmacy, Al-Azhar University, Cairo, 11371, Egypt

**Keywords:** *Suaeda vermiculata*, halophyte, aqueous-ethanolic extract, antioxidant, liver toxicity, cytotoxicity, hepatoprotective, liver disorders, mass spectrometry, LC-MS, HepG-2, HepG-2/ADR

## Abstract

*Suaeda vermiculata*, an edible halophytic plant, used by desert nomads to treat jaundice, was investigated for its hepatoprotective bioactivity and safety profile on its mother liquor aqueous-ethanolic extract. Upon LC-MS (Liquid Chromatography-Mass Spectrometry) analysis, the presence of several constituents including three major flavonoids, namely quercetin, quercetin-3-*O*-rutinoside, and kaempferol-*O*-(acetyl)-hexoside-pentoside were confirmed. The aqueous-ethanolic extract, rich in antioxidants, quenched the DPPH (1,1-diphenyl-2-picrylhydrazyl) radicals, and also showed noticeable levels of radical scavenging capacity in ABTS (2,2′-azino-bis-3-ethylbenzthiazoline-6-sulphonic acid) assay. For the hepatoprotective activity confirmation, the male rat groups were fed daily, for 7 days (*n* = 8/group, *p.o*.), either carboxyl methylcellulose (CMC) 0.5%, silymarin 200 mg/kg, the aqueous-ethanolic extract of the plant *Suaeda vermiculata* (100, 250, and 500 mg/kg extract), or quercetin (100 mg/kg) alone, and on day 7 of the administrations, all the animal groups, excluding a naïve (250 mg/kg aqueous-ethanolic extract-fed), and an intact animal group were induced hepatotoxicity by intraperitoneally administering carbon tetrachloride (CCl_4_). All the animals were sacrificed after 24 h, and aspartate transaminase and alanine transaminase serum levels were observed, which were noted to be significantly decreased for the aqueous-ethanolic extract, silymarin, and quercetin-fed groups in comparison to the CMC-fed group (*p* < 0.0001). No noticeable adverse effects were observed on the liver, kidney, or heart’s functions of the naïve (250 mg/kg) group. The aqueous-ethanolic extract was found to be safe in the acute toxicity (5 g/kg) test and showed hepatoprotection and safety at higher doses. Further upon, the cytotoxicity testings in HepG-2 and HepG-2/ADR (Adriamycin resistant) cell-lines were also investigated, and the IC_50_ values were recorded at 56.19 ± 2.55 µg/mL, and 78.40 ± 0.32 µg/mL (*p* < 0.001, Relative Resistance RR 1.39), respectively, while the doxorubicin (Adriamycin) IC_50_ values were found to be 1.3 ± 0.064, and 4.77 ± 1.05 µg/mL (*p* < 0.001, RR 3.67), respectively. The HepG-2/ADR cell-lines when tested in a combination of the aqueous-ethanolic extract with doxorubicin, a significant reversal in the doxorubicin’s IC_50_ value by 2.77 folds (*p* < 0.001, CI = 0.56) was noted as compared to the cytotoxicity test where the extract was absent. The mode of action for the reversal was determined to be synergistic in nature indicating the role of the aqueous-ethanolic extract.

## 1. Introduction

The liver is a vital organ that regulates both metabolic and detoxification processes. Disorders pertaining to the liver lead to 2 million deaths annually on a global scale [1]. The liver-related mortalities account for the top 15 leading causes of deaths in the United States. In the middle-eastern regions, nearly 10% of the population suffers from the infections of hepatitis-B virus (HBV), and more than 2% of the population is infected with the hepatitis-C virus (HCV), which indicates high prevalence rates of these liver-related infections, which is in addition to other liver-based disorders, including the symptomatic conditions of hyperbilirubinemia-related yellow-coloration, commonly referred to as jaundice. Liver disorders are a serious global healthcare concern [2], and the demand for valuable health-care resources, as well as attention in curtailing them, is huge [3,4]. The common risk factors, such as obesity, diabetes, hypercholesterolemia, and excessive drinking also make the situation further alarming [5]. The available medications for different liver disorders are limited, and some of the currently prescribed chemotherapeutic medications, mainly anti-viral, anti-bacterial, and synthetic steroids, have shown various types of adverse effects in varying proportions to different individuals, and that has the potential to effectively curtail the treatment options, and in turn, negatively impact the desired health outcomes of the subjects [6]. Previous reports on the use of plant-based medications, and investigations of traditionally used herbs, have come back into focus for medicinal purposes, and further drug discovery and development purposes to offer alternative medications to the widely prescribed synthetic-origin drugs for various types of liver disorders. This attention has also provided an impetus to newer strategies in drug development for hepatoprotection purposes [7]. Nonetheless, the use of medicinal plants and their extracts as alternative sources for prophylactic and therapeutic hepatoprotection has always been intriguing, though it has been practiced commonly by traditional healers over time. The majority of the ameliorating claims regarding the hepatoprotective plants, and herbal formulations, remain unproven, vague, and scientifically unsubstantiated, although efforts are still continuously being pushed into the area, and newer plants from varying environments and climatic-condition origins, together with improved testing protocols, are ceaselessly being adopted constantly into the realm of hepatoprotection strategies at global scales [8,9,10,11]. 

Halophytic plants, growing under high-salinity soil conditions, have also been recorded to provide relief from symptomatic liver discomforts [8,9,10]. The halophytes, under the stress of harsh super-saline habitats in areas with the lowest rainfall and marshy conditions, as well as desert environments, have accustomed to adverse environmental conditions through developing various defense mechanisms for survival. These adoptive techniques include multiple strategies which are morphological, biochemical, physiological, metabolic, and sensory in nature [11,12]. One such strategy of these halophytes is their responsiveness to elevated oxidative stress conditions of the plants, wherein these plants survive high-salinity conditions by producing specific enzymatic, non-enzymatic, and metabolite products, many of which are secondary metabolite antioxidant molecules concentrated abundantly in the plant sap [13]. These products have well-established roles in quenching reactive oxygen species (ROS) [14]. The observation that halophytes are rich in antioxidant constituents lends credence to the hepatoprotective claims regarding this category of the plants. The oxidative stress is implicated in the pathogenesis of various other diseases, including liver disorders [15]. The liver, which is also susceptible to acute and chronic toxicities from chemicals, drugs, plant extracts, and nutraceuticals, as well as to harms from an overdose of food components, along with the inherent adverse metabolic and biochemical reactions within the liver, has been damage-controlled and treated by antioxidants, and antioxidant-rich plants, as well as from other natural product extracts rich in antioxidant materials [16,17]. The pharmacological (or intrinsic) toxicity is prevalent over the idiosyncratic toxicity, and this does not have any dose-response or temporal relationship to the generated toxicity [18,19,20]. The ROS are unstable and highly reactive entities and cause the majority of the oxidative-stress related damages and injury to the organs, including the liver, which is comparatively at a higher risk and needs antioxidants to avoid damage and maintain homeostasis. The internally produced glutathione and other enzymatic entities counteract the liver injury caused by the ROS, the reactive nitrogen-based species, and the produced oxidative stress. The oral supplementation of antioxidants for liver disorders has been studied, and several non-enzymatic antioxidants, (e.g., ascorbic acid, α-tocopherol, silibinin, naringenin, quercetin, curcumin, resveratrol, products of phenolic, flavonoid, tannin, and carotenoid types) have been exploited [21,22,23], and the plant-origin secondary metabolite-based antioxidants have revealed promising in vivo therapeutic effects on liver disorders in studied animal models [24], though clinical studies were deemed inconclusive [23]. However, the protective roles of these antioxidants on the liver, in experimentally induced-liver injury and toxication, have been investigated for various pure, single component natural products, and on the naturally-sourced plants’ extracts, as well as mixtures of synergistic natural products containing antioxidants of a structurally-varied chemical nature, and were found to be effective [9,25,26,27,28]. 

In this context, the roles of liver enzymes, and other associated biochemical markers of liver injury and amelioration, are extremely important. The liver’s diagnostic condition is indicated by measuring these enzymes, and markers in the blood and sera samples. Among the major hallmarks of the diseased conditions of the liver are the damage of hepatic cells, and the changed concentrations of antioxidant-natured serum enzymes (i.e., superoxide dismutase, catalase, glutathione peroxidase, and glutathione transferase). The levels of biomarkers in normal and hepatotoxicity-induced animals, albeit in the absence of external antioxidant compounds, and extracts or concoction, differs significantly. The aspartate aminotransferase, alanine aminotransferase, gamma-glutamyl transpeptidase, and alkaline phosphatase enzyme levels, along with the total protein, total bilirubin, and malondialdehyde, are known as liver damage biomarkers present in serum. The normalization of these serum enzyme levels has been observed to signal amelioration of the diseased conditions of the liver [29,30,31,32]. The roles of phenols and polyphenols, flavonoids, tannins, and alkaloid classes of compounds as external antioxidant entities (either as part of the plant-based products, or as a single, pure compound), and the silymarin, a known hepatoprotective agent, are considered crucial in the amelioration process [33,34,35]. The synergistic actions of the enzymatic antioxidants and their source (*S*-adenosylmethionine), as well as the plants’ extract components and individual compounds, have also been reported participating in the liver protection [36,37,38,39,40].

*Suaeda vermiculata*, family Amaranthaceae, is a desert halophyte, renowned for its traditionally claimed liver-protecting activity. This ongoing study aimed to investigate the general claims of the treatment of various symptomatic liver disorders, and proposed to set out to evaluate the safety profile of the plant, given its prevalent use by local herbalists and the public in general. The hepatoprotective activity of the aqueous-ethanolic (aq.-ethanolic) extract from leaves and aerial parts, in addition to the safety of plant material at higher doses, were examined. Studies were carried out to find the antioxidant potential, and concentrations of the antioxidant entities in the aq.-ethanolic extract as being reactive to 1,1-diphenyl-2-picrylhydrazyl (DPPH) and 2,2′-azino-bis-3-ethylbenzthiazoline-6-sulphonic acid (ABTS) in their respective assays. The LC-MS analysis was performed to identify the constituent parts of the aq.-ethanolic extract of the plant and identify the major antioxidants of phenolics and flavonoid nature present in the plant extract. Moreover, the effects of the plant extract on the kidneys, liver, blood sugar, and lipid levels in the CCl_4_-induced toxicity bearing animal models with lipid peroxidation conditions, and liver damages were investigated in detail. The cytotoxic effects of the antioxidant-rich, high-extractive-value aq.-ethanolic extract were also checked on the hepatic cell-lines, HepG-2, and the resistant HepG-2/ADR. 

## 2. Results

### 2.1. LC-MS-Based Chemical Fingerprinting of Suaeda vermiculata aq.-Ethanolic Extract 

The positive mode electron-spray ionization (ESI) analysis of the aq.-ethanolic extract of *Suaeda vermiculata* led to the identification of nine constituents (Table 1). Of these nine identified compounds, three flavonoids were identified in the plant extract: One flavonol aglycone (quercetin), and two flavonol glycosides (quercetin-3-*O*-rutinoside or rutin, and kaempferol-*O*-(acetyl)-hexoside-pentoside). 

### 2.2. Estimations of the Total Phenolics and Flavonoid Contents in the aq.-Ethanolic Extract, and Fractions of the aq.-Ethanolic Extract Obtained from Suaeda vermiculata

The total phenolics and flavonoid contents of the aq.-ethanolic extract of *Suaeda vermiculata* and its fractions were estimated as gallic acid (GAE) and quercetin equivalents (QE) (Table 2). A significant amount of total phenolics contents were present in the aq.-ethanolic extract (200.6 ± 2.14 mg GAE/g of the extract’s dry weight). However, the ethyl acetate fraction possessed comparatively very high concentrations of total phenolics (317.5 ± 0.17 mg GAE/g of fraction’s dry weight). The majority of the flavonoids’ contents were determined to be present in the chloroform fraction (17.47 ± 0.02 mg QE/g of fraction’s dry weight), followed by the aq.-ethanolic extract (16.70 ± 1.56 mg QE/g of the extract’s dry weight).

### 2.3. Determination of Antioxidant Potentials Through Measurement of Free-Radical Scavenging Activity

**Figure 1:**
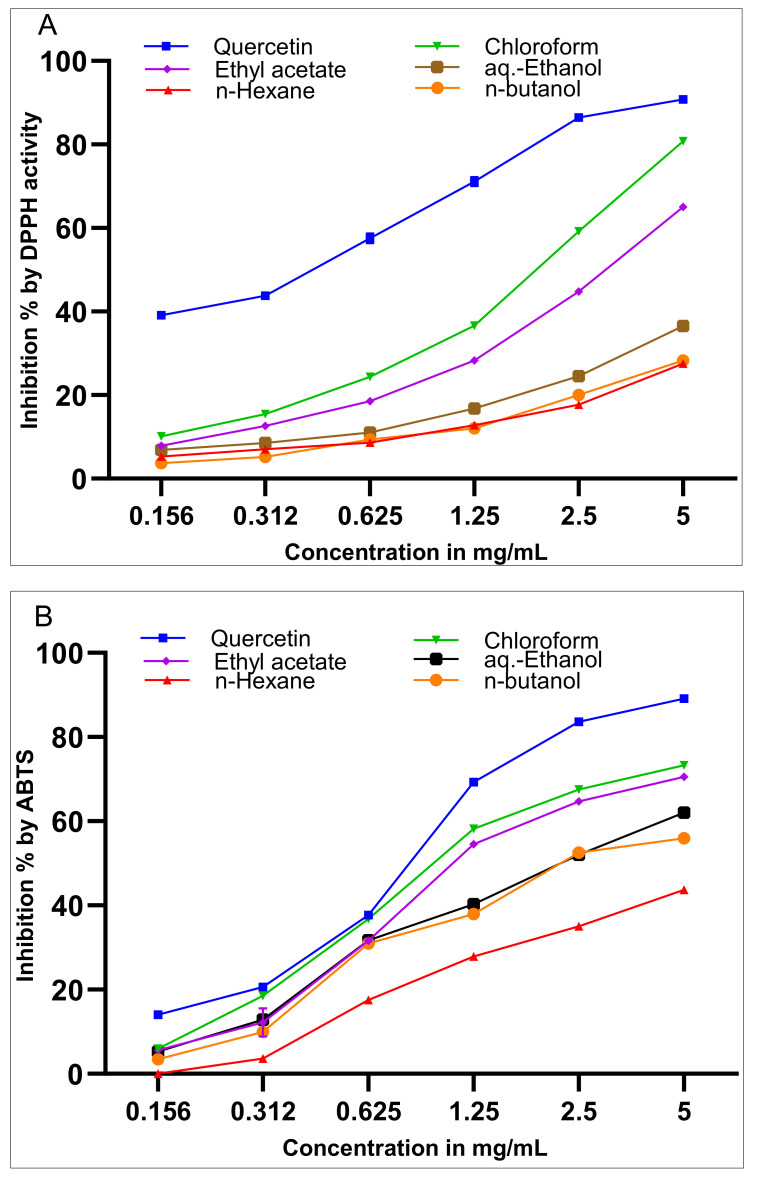
Radical scavenging activity of *Suaeda vermiculata* extracts by (**A**) 1,1-diphenyl-2-picrylhydrazyl (DPPH), and (**B**) 2,2’-azino-bis-3-ethylbenzthiazoline-6-sulphonic acid (ABTS) methods. Values are the mean of three replicates ± SEM. *p* < 0.0001 using two-way ANOVA, and *p* < 0.0001 compared to quercetin for all groups at all the measured concentrations for both methods according to multiple comparisons for Tukey’s method.

The in vitro radical scavenging activity of the *Suaeda vermiculata* mother liquor aq.-ethanolic extract and its subsequent fractions were estimated by the DPPH and ABTS radical scavenging methods, and IC_50_ values (concentrations causing 50 % inhibitions) were determined based on the requisite percentage inhibitions versus the concentrations (Figure 1). The chloroform and ethyl acetate fractions demonstrated significant concentration-dependent DPPH and ABTS scavenging activities, all being maximum at 5 mg/mL as compared to the standard quercetin at the same concentration (Figure 1). The lowest antioxidant activity was observed for the *n*-hexane fraction in scavenging the DPPH and ABTS free radicals, followed by the antioxidant activity of the *n*-butanolic fraction. The DPPH radical quenching-based antioxidant activity levels were also recorded for the chloroform, and ethyl acetate fractions, observed at about 80 and 65 % of strengths, respectively, while the mother liquor, aq.-ethanolic extract, showed nearly 40 % DPPH radical inhibition strength, all as compared to the referral standard, quercetin (Figure 1A). The ABTS free radical scavenging antioxidant activity was also assayed for the chloroform and ethyl acetate fractions and was found at 72 and 70 % strengths, respectively, followed by the aq.-ethanolic extract antioxidant activity levels at 60 % antioxidant strength in comparison to the standard quercetin (Figure 1B). 

### 2.4. Acute Toxicity Study and Dose Selection 

Among all the groups (50, 300 mg/kg; 2 and 5 g/kg) checked for acute toxicity, one aq.-ethanolic extract-treated rat (2 g/kg) died on day 2 of the dose administration. The death was attributed to a gavage accident since no behavioral pattern difference was observed among the extract-fed or the control group. According to the Organization for Economic Cooperation and Development (OECD) guidelines, the dose is considered toxic if more than two animals die. All the animal groups were monitored daily for any visible signs of toxicity and mortality for up to two weeks. None of the remaining animals in all the groups displayed any visible signs of toxicity, such as anorexia, hair erection, lacrimation, tremors, convulsions, salivation, or diarrhea, in the first 24 h until 2 weeks of the study period. Based on the data, the given dose was determined to be practically non-toxic according to Hedge and Sterner scale, and 10% (500 mg/kg) of the maximum administered dose (5 g/kg) in the acute toxicity study was selected as the maximum dose for the pharmacological evaluation [49].

### 2.5. Safety Determination of aq.-Ethanolic Extract on Liver, Heart, and Kidney

The administration of the aq.-ethanolic extract of *Suaeda vermiculata* (250 mg/kg, CCl_4_) in normal rats showed no significant effects on the levels of liver enzymes AST (aspartate transaminase) and ALT (alanine transaminase) and was significantly lower (*p* < 0.02) for TP (total protein) contents estimation compared to the intact control group. Moreover, the serum creatinine, triglycerides, cholesterol, and glucose levels showed no significant differences in the 250 mg/kg, CCl_4_- group when compared with the intact control group of animals (Table 3 and Table 4). 

### 2.6. Hepatoprotective Activity of the aq.-Ethanolic Extract

**Figure 2:**
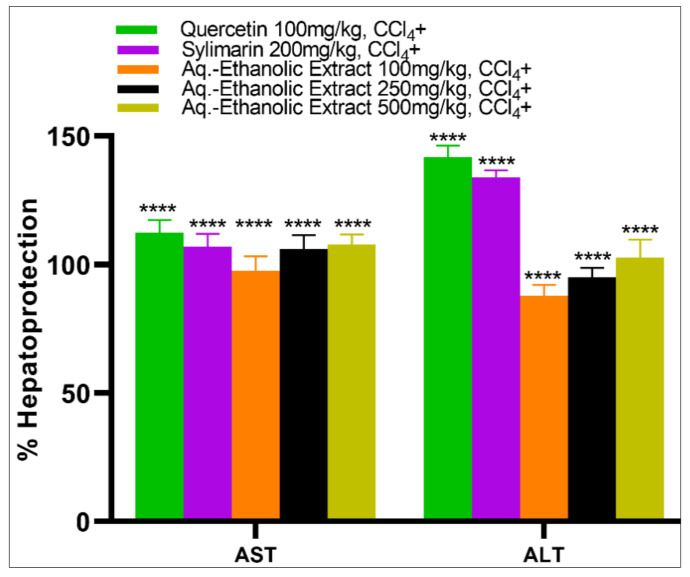
The hepatoprotective effects of *Suaeda vermiculata* aq.-ethanolic extract on CCl_4_ induced liver toxicity. Percentage protection of CCl_4_ induced elevation of AST and ALT enzymes. The protection percentage formula assumes the enzyme level of the CCl_4_ administered negative group (carboxyl methylcellulose) at 0% protection and that of intact group (no exposure to CCl_4_) at 100% protection as such they are excluded from the graphical representation. In addition, the aq.-ethanolic extract 250 mg/kg CCl_4_- the group which did not receive CCl_4_- is also excluded from the graphical representation. **** Data differed significantly at *p* < 0.0001 when compared with the negative control group. Values are expressed as mean ± SEM, *n* = eight rats per group, CCl_4_-: Carbon tetrachloride was not administered. CCl_4_+: Carbon tetrachloride-induced liver toxicity.

The AST and ALT values for the CCl_4_-induced negative control group treated with CMC were significantly higher (*p* < 0.0001) when compared with all the CCl_4_+ induced hepatotoxic groups, i.e., intact, silymarin (200 mg/kg), quercetin (100 mg/kg), and the extract-fed groups (100, 250, and 500 mg/kg) (Table 3). Animals were given *Suaeda vermiculata* aq.-ethanolic extract at daily doses of 100, 250, and 500 mg/kg, and showed a dose-dependent decrease in AST and ALT liver enzyme markers. The 500 mg/kg dose-fed animal group showed maximum hepatoprotective activity (Figure 2), and this group of animals effectively prevented the CCl_4_-induced elevation of serum enzyme markers of ALT and AST as compared to the CMC group (CCl_4_ induced). The silymarin (200 mg/kg), and quercetin (100 mg/kg)-fed animal groups also showed a significant decrease in AST and ALT liver marker enzymes, while the silymarin group also showed significantly decreased TP contents as compared to the negative control group of animals. No differences were found in the levels of creatinine, or glucose in any CCl_4_+ induced hepatotoxic groups pre-treated with various doses of aq.-ethanolic extract as compared to the negative group (Table 3 and Table 4). The cholesterol level of the aqueous-ethanol extract-fed group at 250 mg/kg was comparable to the intact control. The extract doses of the 100 and 500 mg/kg-fed animal groups caused a significant decrease in the triglyceride levels as compared to the negative control group of animals (Table 4). 

### 2.7. Cytotoxicity of Suaeda vermiculata Extract in Wild Type Sensitive and Resistant Hepatic Cell-Lines

**Figure 3:**
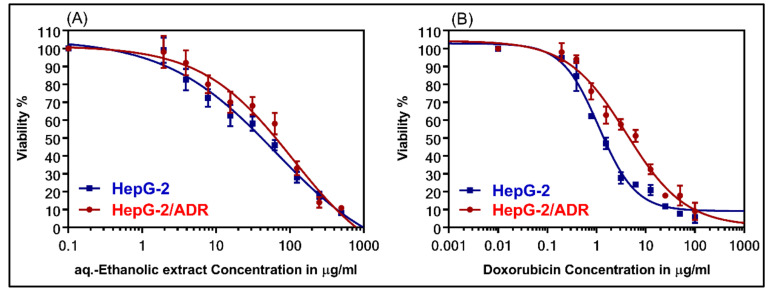
The dose-response curves of *Suaeda vermiculata* aq.-ethanolic extract: (**A**) aq.-ethanolic extract, and (**B**) doxorubicin (DOX) in wild-type HepG-2, and resistant HepG-2/ADR cell-lines.

To test the cytotoxicity of *Suaeda vermiculata* aq.-ethanolic extract in sensitive HepG-2 and resistant cells, HepG-2/ADR, evaluations were conducted in comparison to the standard cytotoxic drug, doxorubicin (DOX). Figure 3 demonstrates the dose-response curves of the *Suaeda vermiculata* aq.-ethanolic extract group in comparison to the chemotherapeutical agent, DOX. The moderate cytotoxic effect of *Suaeda vermiculata* aq.-ethanolic extract in HepG-2 showed an IC_50_ value of 56.19 µg/mL, and in HepG-2/ADR-resistance cells, the IC_50_ value was 78.40 µg/mL with relative resistance (RR) at 1.39 (Table 5).

### 2.8. Suaeda vermiculata aq.-Ethanolic Extract Reverses DOX Cytotoxicity in Resistant Hepatic Cell-Lines

**Figure 4:**
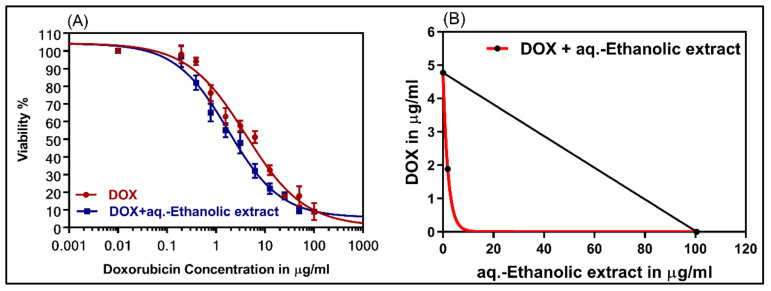
(**A**) The dose-response curves and isobologram analysis of the combination of DOX with 20 µg/mL of *Suaeda vermiculata* extract in resistant HepG-2/ADR. (**B**) The isobologram on the right side of the figure showed the synergistic interaction between DOX and the extract. IC_50_, DOX, and IC_50_ extract correspond to the IC_50_ for DOX and extract alone. CI < 1 indicates synergism, CI = 1 indicates additive, and CI > 1 indicates antagonism.

To examine the reversal effects of the combination of DOX and *Suaeda vermiculata* aq.-ethanolic extract, the DOX-resistant HepG-2/ADR cell lines were selected. The DOX-resistant cells were treated with DOX in the presence and absence of 20 µg/mL *Suaeda vermiculata* aq.-ethanolic extract. As shown in Figure 4, *Suaeda vermiculata* extracts synergistically enhanced the cytotoxicity of DOX, as observed from the dose-response curves of the combination and isobologram. The IC_50_ values of DOX alone or in combination with *Suaeda vermiculata* aq.-ethanolic extract in DOX resistant cells are shown in Table 6. The combination of DOX with *Suaeda vermiculata* extract significantly reduced (*p* < 0.001) the IC_50_ value of DOX, with FR in resistant cell lines to 2.77, and CI at 0.56 (Table 6). 

## 3. Discussion

The identification of the flavonoid derivatives (quercetin, quercetin-3-*O*-rutinoside or rutin, and kaempferol-*O*-(acetyl)-hexoside-pentoside) in the aq.-ethanolic extract of the *Suaeda vermiculata* is consistent with the halophytic nature of the plant, thereby having excessive antioxidants compounds, especially flavonoids. In addition, the nature of these flavonoids is in conformity with the previously isolated and identified flavonoids from the genus *Suaeda* [43]. For instance, the quercetin (12.45%), and quercetin-3-*O*-rutinoside (7.80%) were previously isolated from *Suaeda japonica, Suaeda salsa,* and *Suaeda physophora* [43], while kaempferol and kaempferol glycoside are also common constituents of the genus *Suaeda* plants, and have been reported both from *Suaeda japonica* and *Suaeda asparagoides* [43]. The LC-MS analysis of the aq.-ethanolic extract showed a base peak at *m/z* 872.1408 [M + H]^+^ which was identified as the pheophytin-a (23.69%), the reported major chlorophyll-based constituent from *Suaeda vermiculata* species [48]. The fragmentation pattern for pheophytin-a showed mass peaks at *m/z* 256.26268 [phytyl-C_2_H_5_ + H], 280.26241 [phytyl moiety], 593.15607 [M-phytyl], 577.12459 [M-phytyl–methyl], and *m/z* 615.13623 [M-phytyl + Na], which together confirmed the structure of the compound. The LC-MS analysis of the plant’s aq.-ethanolic extract also showed base peaks for the rhein (anthraquinone aglycone) at *m/z* 285.04069 [M + H]^+^, in addition to the base peaks for senecic acid, 4-acetyl-6-(2-methylpropionyl)-1,3-resorcinol, (2E)-heptyl-3-(3,4-dihydroxyphenyl) acrylate, and hexadecanedioic acid at *m/z* 217.10656 [M + H], 223.09634 [M + H]^+^, 279.15900 [M + H]^+^, and *m/z* 287.22140 [M + H], respectively. 

The higher contents of phenolics in the ethyl acetate fraction is probably owing to the ability of ethyl acetate as a mid-polarity solvent to dissolve the phenolic contents of the aq.-ethanolic extract much easier than the relatively non-polar, and lipophilic solvents, i.e., *n*-hexane, and chloroform. Besides, the ethyl acetate fraction accumulated more phenolics and flavonoid contents compared to the *n*-butanol fraction, which could be due to the fact that ethyl acetate as a solvent was used in the extraction sequence of aq.-ethanolic mother liquor before the *n*-butanol. Additionally, the majority of the flavonoid contents were present in the chloroform fraction (17.47 ± 0.02 mg QE/g of fraction’s dry weight), which was closely followed by the aq.-ethanolic extract (16.70 ±1.56 mg QE/g of extract’s dry weight), indicating a lesser degree of hydrophilicity of the products, culminated through the presence of fewer hydroxylation sites and glycosylation of the flavonoids encountered in the chloroform extract, which were able to be extracted in this lipophilic solvent under the presence of aqueous partitioning phase. 

The presence of higher concentrations of phenolics and flavonoids is consistent with the previously published data about flavonoid and phenolic contents present in the solvents, such as chloroform and ethyl acetate [50,51,52,53]. In the current study, the relatively high mg per gram content values of the phenolics and flavonoids are in the chloroform and ethyl acetate fractions compared to the mother liquor and confirms the higher antioxidant activity of these fractions over the mother liquor. Furthermore, the pheophytin-a, which was reported for the mild antioxidant activity, and isolated previously form *Suaeda vermiculata* chloroform fraction [48] as part of the ongoing study, also played a role in the antioxidant activity of the chloroform fraction. Additionally, the steroidal constituent, β-sitosterol [48], along with other unsaturated oxygenated products [54] identified in the chloroform fraction of the *Suaeda vermiculata* contributed to the higher antioxidant value of the chloroform fraction. Nonetheless, the mother liquor aq.-ethanolic extract was pursued in further studies into the biological evaluations as a hepatoprotective extract, since the traditional practice of the use of plants’ aerial parts as a whole for liver disorders provided an equivalence between the prevailing practice and the aq.-ethanolic extract, the mother liquor obtained from the plant’s extraction. The mother liquor aq.-ethanolic extract was also selected for the biological evaluations as it represented the largest number of constituents obtained through a comparatively significant high-extractive value (20 g/100 g of the dried plant herbs). Moreover, the statistically insignificant difference (Table 2) in the flavonoid contents of the aq.-ethanolic extract (16.70 ± 1.56 mg/g QE) in comparison to the chloroform fraction’s flavonoid contents (17.47 ± 0.02 mg/g QE) supported the choice of the aq.-ethanolic extract for biological activity evaluations.

**Figure 5:**
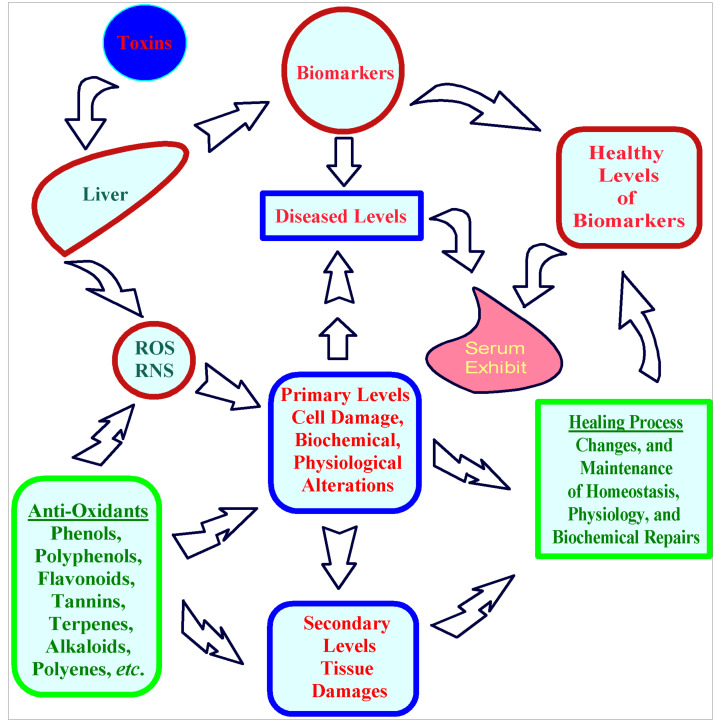
Prospective roles of antioxidants in hepatoprotection.

The current investigation confirmed the *Suaeda vermiculata* plant’s richness in antioxidant components compared to other *Suaeda* species [43]. The in vitro radical scavenging activity of the *Suaeda vermiculata* mother liquor aq.-ethanolic extract and its subsequent fractions were estimated by the DPPH and ABTS radical scavenging methods, and the IC_50_ values (concentrations causing 50% inhibitions) were determined based on the requisite percentage of radicals’ inhibitions versus the concentrations (Figure 1). Notably, the presence of higher concentrations of antioxidant compounds, measured as radical scavenging capacity in DPPH and ABTS assays is a direct indication of the antioxidant potential of the plant’s constituents, but not necessarily the high presence of phenolics and flavonoids, which can only be the reasons for higher antioxidant potential, though these two categories of products, i.e., phenolics and flavonoids, seem to be the reason for the dominating antioxidant potential of the plant. The higher antioxidant presence in the extract and the fractions of the *Suaeda vermiculata* can be attributed to the high-salinity habitat of the plant in comparison to its other counterpart species growing in different climatic locations [43]; the former has been the herb of choice for the local population for treatment of liver disorders, especially jaundice [48,55]. However, the biomechanistics connections between the higher antioxidant concentration levels of compounds in the plant, and its hepatoprotective action have not been conclusively established, though it has been well-observed, and a relationship is proposed at the primary levels (Figure 5), albeit without intricate and basic biomechanistics roles playing, and interconnections, or the feedback responsibilities of the components in the presented cycle. Nonetheless, a plausible mechanistic explanation of the toxicity and its amelioration, based on known information, is proposed herein (Section 3.1). 

Moreover, it has to be noted that the present study found no adverse effects of the aq.-ethanolic extract on the liver and kidney enzymes of the normal rats, and the plant extract also did not induce any toxicity at doses as high as 5 g/kg. The study also revealed that the aq.-ethanolic extract of *Suaeda vermiculata* had no significant effects on the blood glucose levels for the treated normal rats as compared to the control group of animals which (blood glucose levels) was measured at 101.82 ± 4.47 and 122.46 ± 2.66 mg/dL, respectively. Additionally, the natural healing in CCl_4_ -induced hepatotoxic animals, ingestion of potent hepatotoxins, and evaluations of the hepatoprotective action using several natural products are abundantly available in the literature [56,57,58]. As for the local *Suaeda* species, where the *Suaeda vermiculata* is concerned, there were no other pharmacological data of any kind, comparative, or otherwise available on the liver-protecting activities of this plant or the plants of the differently-located *Suaeda* species, referring to those located in Iran. 

The locally procured *Suaeda vermiculata* aq.-ethanolic extract, as shown in Table 5, exhibited moderate cytotoxicity on HepaG-2 (IC_50_ 56.19 ± 2.55 µg/mL), designated in accordance with the provisions of the previous classification of the cytotoxicity criteria of the natural products extracts on cell lines [59]. As previously reported, the extract was considered active if it had a mean IC_50_ value <100 µg/mL; a moderate level of activity was considered between 10–100 µg/mL; and the strong cytotoxic activity was designated with <10 µg/mL of the IC_50_ values. *Suaeda vermiculata* aq.-ethanolic extract also exhibited moderate cytotoxicity in HepG-2/ADR, the resistance cell-lines (IC_50_ 78.40 ± 0.32 µg/mL with RR 1.39) (Table 5). In resistance cell lines, like HepG-2/ADR, the P-gp is overexpressed as part of the mechanism to escape cell-death induced by anti-cancer chemotherapeutic agents. The cytotoxicity results indicated that *Suaeda vermiculata* aq.-ethanolic extract could be a potential modulator of P-gp, most probably due to the presence of quercetin and other flavonoids (kaempferol, and rutin, Table 1). The flavonoids and phenolics, e.g., kaempferol, rutin, apigenin, daidzein, fisetin, luteolin, silibinin, naringin, proanthocyanidin, (-)epicatechin-3-*O*-gallate, etc., are known P-gp inhibitors [60,61,62]. The *Suaeda vermiculata* aq.-ethanolic extract’s cytotoxicity results were further confirmed from a combination experiment which improved the cytotoxicity of DOX (Table 6) through combination with a low and non-toxic concentration (20 µg/mL) of the aq.-ethanolic extract [63,64]. Again, the presence of the phenolics, together with flavonoids components in the aq.-ethanolic extract (Table 1) could explain these inhibitory effects [63].

### 3.1. A Plausible Biomechanistic Aspect of Liver Toxicity and Its Amelioration

The induction of liver toxicity by carbon tetrachloride (CCl_4_) ensues through free radicals generation [65]. The cytochrome P450 bio-transforms CCl_4_ [66] to form trichloromethyl radical (CCl_3_•), which, in turn, in the presence of oxygen, lipid, and protein, forms trichloromethyl peroxyl radical (CCl_3_OO•) that eventually leads to lipid peroxidation [67]. These transformations lead to hepatocyte dysfunction through the destruction of the intracellular and the plasma membranes of the organ [68,69,70]. 

**Figure 6:**
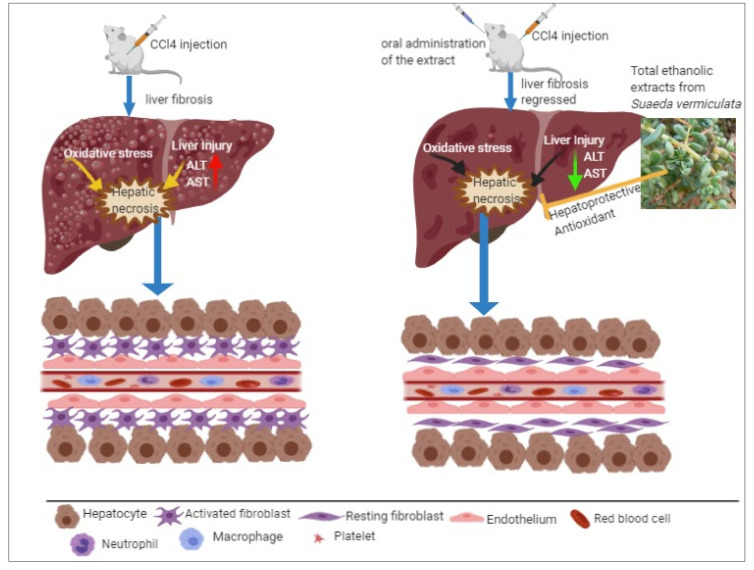
Sketch representing the role of the plant extract in liver protection.

The hepatotoxicity was induced in animals by CCl_4_ injections and led to an increase in serum aminotransferase AST and ALT levels, generally being an AST > ALT relationship in the toxic situations [71], wherein both the AST and ALT were considered as biomarkers of liver damage [72,73,74,75]. In the current study, rats with CCl_4_-induced hepatotoxicity were confirmed to have significantly increased levels of AST and ALT in the negative control compared to the intact group of animals (Table 3). The administration of the aq.-ethanolic extract induced significant (*p* < 0.05) hepatoprotective action against CCl_4-_induced liver-toxicity by improving the liver functions, as indicated by the reductions in the levels of the liver enzymes, ALT, and AST, again as compared to the negative control (Table 3). The aq.-ethanolic extract-treated animals showed liver enzyme levels at comparable levels to the intact animals (as standard controls), and silymarin and quercetin groups of animals. Silymarin is an established antioxidant also used as a standard referral compound in the evaluation of the hepatoprotective activity of nutraceuticals, and other natural products [76,77,78]. The protective effects of quercetin are associated with a decrease in the oxidative stress in the hepatotoxicity-induced liver tissues [79,80]. The AST values for all the aq.-ethanolic extract dose groups in the current study are comparable with the quercetin. The aq.-ethanolic extract showed protective effects in CCl_4_-induced hepatotoxicity conditions, as well as safety from toxicity induction up to 5 g of the aq.-ethanolic extract administration, which showed no adverse effect on the extract-fed animals. The protective effects of the aq.-ethanolic extract indicated the ameliorating effects of the *Suaeda vermiculata* concerning the liver cells in the in vivo conditions (Figure 6).

The comparatively higher concentrations of the flavonoids and phenolics in the halophytes, in comparison to the other normal-conditioned plants, might also be the scientific explanation for their liver-protecting activity [43,48,54,81,82]. Moreover, the hypoglycemic effects reported from other plants of the genus *Suaeda*, for example, *Suaeda fruticosa* produced a significant decrease in the blood glucose levels in normal rats, and a further reduction in the diabetic rat models was observed [83]. The study reported that the aqueous extract of *Suaeda fruticosa* induced a significant decrease in the plasma cholesterol without noticeably affecting the plasma triglyceride levels in the treated rats. The free-radical scavenging potential and the inter-linked antioxidant components in the plant extract reportedly played a critical role in hepatotoxicity which was mediated by the actions of free-radicals on hepatocytes leading to cell necrosis [81]. In this connection, the *Suaeda vermiculata* aq.-ethanolic extract also showed free-radical inhibition properties, and the data reported in this study is following previously published reports demonstrating the hepatoprotective effects in CCl_4_-induced extensive liver damage models by the aq.-ethanolic extract containing antioxidant compounds as part of the mother liquor obtained from the plant [56,57,58,66,84,85,86,87,88,89,90]. 

## 4. Materials and Methods

### 4.1. Chemicals and Reagents

All chemicals were of analytical grade. Methanol (HPLC grade), carbon tetrachloride, formic acid, (Sigma Aldrich, USA), doxorubicin was also of analytical grade. Silymarin tablets were obtained from Micro Labs Limited, Mumbai, India, and used as obtained.

### 4.2. Plant Material Collection and Extraction 

The *Suaeda vermiculata* plant material (2 kg) was collected (10/2019) from Buraydah City, Qassim, and identified by the institutional botanist at Qassim University (QU). The shade-dried herbs were grinded to a coarse powder (1.4 kg), and extractions performed following the cold maceration method under stirring for 24 h from 70% aqueous-ethanol, repeatedly (3Lx3). The hydroalcoholic extract was filtered and evaporated to dryness under reduced pressure and <40 °C temperature to yield 86.3 g of the dried extract, part of which was stored under −20 °C for further use. The dried aq.-ethanolic extract (50 g) was suspended in 500 mL distilled water and fractionated with *n*-hexane, chloroform, ethyl acetate, and *n*-butanol. The fractions were subjected to dryness under vacuum and <40 °C, and stored under −20 °C for future use. 

### 4.3. LC-MS Experimental Design

Chemical fingerprinting of the aq.-ethanolic extract was performed by C_18_ reverse phase HPLC chromatographic analysis. The compounds were identified by the coupled MS spectrometry through data interpretation and were compared with the literature-reported mass values. The compounds’ identification processes were based on the data from the internal library software, and comparison of the available mass-fragments with the literature reports. The relative percentages of the identified compounds were calculated in relation to the total area of the chromatogram. Analytical results are summarized in Table 1. 

#### 4.3.1. High-Pressure Chromatography (HPLC): The Chemical Fingerprinting

The separation procedure was carried out using a C_18_ column (C_18_ synchronism 250 × 2.1, 5 µ). The mobile phase solvents were composed of A: 100% water + (0.1 % formic acid), and B: 100% methanol + (0.1 % formic acid). The gradient elution program was followed. The injections [methanol blank, caffeine (QC), and the samples] volume was 20 µL for each, and the flow rate was set at 450 µL/min. UV-Vis detection was performed, and the scanned wavelength was set between 220–600 nm with a scan rate of 20 Hz. Two channels (254 and 268 nm) were also used. The XcaliburTM software (Thermo Scientific) was used for method development and data treatments. All the chromatographic work was carried out at the Faculty of Medicine Laboratories, Umm Al-Qura University, Makkah, Saudi Arabia. 

#### 4.3.2. Mass Spectrometer

The analysis was performed using a Thermo LTQ Velos Orbitrap mass spectrometer (Thermo Scientific, Pittsburgh, PA, USA) equipped with a heated ESI ion source. The mass scan range was set to 100–2000 *m/z*, with a resolving power of 100,000. The *m/z* calibration of the LTQ-Orbitrap analyzer was performed in the positive ESI mode using a solution containing caffeine, MRFA (Met-Arg-Phe-Ala) peptide, and Ultramark 1621, in accordance with the manufacturer’s guidelines. The ESI was operated with a metal needle operating at 4.5 kV. For all the experiments, the source vaporizer temperature was adjusted to 450 °C, the capillary temperature was set at 275 °C, and the sheath and auxiliary gases were optimized, and set to 30 and 15 arbitrary units, respectively.

### 4.4. Determination of Total Phenolic Contents

The total phenolic contents of the extract and fractions were determined using the Folin–Ciocalteu method [91]. Briefly, 4 mL of the extract/fraction (40 µg/mL in ethanol) was mixed with 2.5 mL of the Folin–Ciocalteu reagent (diluted with distilled water, 10%, *v/v*) for 5 min, and then 2.5 mL of 20% (*w/v*) sodium carbonate was added. The mixture was allowed to stand for 60 min in the dark, and the absorbance was measured at 760 nm using a V-630, JASCO, Japan, UV-Visible spectrophotometer. The analyses were performed in triplicate, and the total phenolic contents were calculated using the linear regression equation of the calibration curve (y = 0.002x − 0.014, R² = 0.998) of gallic acid performed between the concentrations of 0.05 to 0.5 mg/mL, and expressed as the mg of gallic acid equivalent per gram (mg GAE/g) of the dried extract/fractions weights (Table 2).

### 4.5. Determination of Total Flavonoid Contents

The total flavonoid contents were estimated using the aluminium chloride complex-forming assay [92]. An aliquot of extract/fraction (1.5 mL of 1 mg/mL in ethanol) was mixed with 500 µL of 10% aluminum chloride solution, and the mixture was allowed to stand for 60 min. Absorbance was measured at 510 nm, and a calibration curve (y = 0.001x + 0.013 R² = 0.994) for quercetin was then plotted using 0.05 to 0.5 mg/mL of quercetin in methanol. The equivalents of milligram quercetin per gram (mg QE/g) of the fully concentrated extract/fractions were used to calculate the total flavonoid contents.

### 4.6. Evaluation of Free-Radical Scavenging Activity 

#### 4.6.1. DPPH Assay

The scavenging activity of the extract was estimated using 1,1-diphenyl-2-picrylhydrazyl (DPPH) as a free-radical [93] with slight modification. The DPPH working solution (1.5 mL, 100 µM in methanol) was mixed with 0.5 mL of the extract/fraction/standard (quercetin solution) at different concentrations (0.156 to 5 mg/mL). The mixtures were vortexed thoroughly for 5 min and left in the dark at room temperature (RT) for 20 min, and absorbance was measured at 517 nm to estimate the reduction in DPPH color intensity. The scavenging activity of the extract/fraction and standard control were calculated using the following equation:

(1) $$\;\mathrm{Scavenging}\;\%=\left(1-\frac{S_{\mathrm{ab}}}{B_{\mathrm{ab}}}\right)\times 100\;$$

The **S**_ab_ and B_ab_ refer to the sample absorbance and blank absorbance, respectively.

#### 4.6.2. ABTS Assay

The ability of the extract and fractions to scavenge ABTS radical formation was assayed according to the method of Floegel et al. [94] with slight modification. The radical cation of the ABTS (2,2’-azino-bis-3-ethylbenzthiazoline-6-sulphonic acid) was generated by heating the mixture of 2,2-azobis(2-amidinopropane) dihydrochloride (1 mM) and 2.5 mM of the ABTS in phosphate buffer (10 mM, pH 7.4) at 68 °C for 40 min on a water bath. The mixture was allowed to cool to room temperature and filtered. The freshly prepared ABTS solution (980 µL) was mixed with 20 µL of the serially diluted extract/fraction, and quercetin solution (0.156 to 5 mg/mL in ethanol) separately. The mixture was incubated for 10 min at 37 °C before the measurement of ABTS color reduction at 734 nm using a spectrophotometer. The ABTS scavenging activity of the extract and standard quercetin was calculated by defining the percentages of the absorbance reduction of ABTS-extract mixture in comparison to the absorbance of the ABTS blank solution from the following equation:

(2) $$\mathrm{Scavenging}\;\%=\left(1-\frac{T_{\mathrm{ab}}}{C_{\mathrm{ab}}}\right)\times 100\;.$$

The T_ab_ and C_ab_ refer to the sample and blank absorbances, respectively.

### 4.7. Acute Toxicity Studies and Sample Size

Acute toxicity studies were conducted following the OECD procedure for acute toxicity testing [95,96]. Briefly, ten-week-old female Sprague Dawley rats (*n* = 20), weighing 200 ± 50 g, overnight fasted, were randomly divided and weighed, and single aq.-ethanolic extract doses were administered (*n* = 5/group) using oral (p.o.) route with 50 and 300 mg/kg, and 2 and 5 g/kg. The animals were observed for abnormality in behavior and movement for the first three days and mortality for up to two weeks [96]. 

The required sample size for the experiment was determined using mean ± SEM AST values between the CCl_4_-induced injury untreated group and the CCl_4_-induced injury aq.-ethanolic extract-treated group, based on a previously published report [58]. The calculated effect size d was 4.27 using a two-tail option on G Power V.3.1.9.4 software, Heinrich Heine University, Düsseldorf, Germany [97]. To achieve a statistical power (1-β err prob) of 80 % and a specific α error probability of 0.05, the minimum required sample size in each group was *n* > 3 mice.

### 4.8. Effect of aq.-Ethanolic Extract on Liver and Kidney Enzymes in Addition to Lipid and Sugar Levels

The study was performed in accordance with the Animal Research: Reporting In Vivo Experiments (ARRIVE) statement [98].

#### 4.8.1. Experimental Animal Groups

Male ten-week-old naïve Sprague Dawley rats (*n* = 64) weighing 200 ± 50 g, obtained from the animal house facility, College of Pharmacy, Qassim University, Saudi Arabia, were maintained in individual polyacrylic cages housing 2 or 3 rats with a chow diet obtained from the First Milling Company in Qassim, Buraydah, Saudi Arabia, and water ad libitum, 7 days before the start of the experiments. The animals were maintained at RT (~ 25 °C) and relative humidity of ~65% with a controlled light-dark cycle of 12:12 h. The institutional Research Ethics Committee approved the experimental procedure and animal care (Approval ID 2019-CP-8) as per the Guidelines for the Care and Use of Laboratory Animals. Animals were divided randomly into eight groups (*n* = 8/group). The intact animals (group I) remained untouched during the experimental procedure, while another group received 250 mg/kg (group II) of the aq.-ethanolic extract of *Suaeda vermiculata* orally for seven days to monitor the effect of the extract on the liver and kidney. The remaining groups were administered p.o. once daily with carboxyl methylcellulose 0.5% (CMC, negative control, group III), 200 mg/kg silymarin (positive control, group IV) [99,100,101], or aq.-ethanolic extract dose of 100, 250, or 500 mg/kg (groups V–VII), and 100 mg/kg quercetin (group VIII) [102,103] for 7 days, followed by induction of hepatotoxicity using single intraperitoneal (i.p.) dose of CCl_4_ dissolved in olive oil (1:1, 1.0 mL/kg) [56]. Olive oil (an emulsifying agent) helped to dissolve CCl_4_ sufficiently for induction of liver damage and is neither toxic nor possesses any hepatotoxicity related to pharmacological activity [57,58,104]. Twenty-four hours after the administration of CCl_4_, blood samples were withdrawn from the orbital vein in ethylene diamine tetraacetic acid (EDTA)-heparinized tubes under mild general anesthesia with ketamine, and samples were centrifuged to separate plasma [105]. The separated plasma was used for the determination of levels of aspartate transaminase (AST) and alanine transaminase (ALT) levels, total protein (TP), creatinine, blood glucose, total cholesterol, and triglycerides with spectrophotometric assays. Percentage hepatotoxic protection was calculated using the following equation [106]:

(3) $$\mathrm{Hepatoprotection}\;\%\;=\left[\frac{a-b}{a-c}\right]X\;100$$

where a, b, and c refer to the mean value of the marker enzyme level produced by hepatotoxin; toxin plus test sample, and control, respectively. 

#### 4.8.2. Determination of Levels AST, ALT, TP, and Creatinine

The levels of ALT and AST in plasma samples were determined using an optimized UV-test, in accordance with the International Federation of Clinical Chemistry (IFCC) procedure. The kit was supplied by the Crescent Diagnostics Company, KSA, (product catalog #CZ902L for ALT, and #CZ904L for AST). The absorbances were measured at 340 nm, and the readings were multiplied by a factor of 952 using the kinetic method [107]. The plasma level of TP was measured using the photometric, colorimetric test. The cupric ions reacted with protein in an alkaline solution to form a purple complex. The absorbance of the complex was proportional to protein concentration in the samples [108]. The plasma creatinine was determined by the kinetic method without deproteinization–Jaffe reaction (Crescent Diagnostics Company, #604). In the Jaffe reaction, the creatinine reacted with alkaline picrate to produce a reddish-orange color, the intensity of which at 490 nm was directly proportional to creatinine concentration [109]. 

#### 4.8.3. Determination of Plasma Level of Glucose, Cholesterol, and Triglycerides 

The glucose level was determined after enzymatic oxidation in the presence of glucose-oxidase (Crescent Diagnostics Company, #605). The formed hydrogen peroxide reacted under the catalysis of peroxidase with phenol and 4-aminoantipyrine to form a red-violet using quinone imine dye as an indicator [110]. The total amount of cholesterol was determined after enzymatic hydrolysis and oxidation (Crescent Diagnostics Company, #603). The colorimetric indicator quinone imine was generated from phenol and 4-aminoantipyrine by hydrogen peroxide under the catalytic action of peroxidase, and the color intensity was then measured at 546 nm [111]. The triglycerides were determined after enzymatic splitting with lipoprotein lipase (Crescent Diagnostics Company, #611) wherein the indicator was quinone imine, generated from 4-chlorophenol and 4-aminoantipyrine by hydrogen peroxide to form a red color quinone imine dye measured at 546 nm [111].

### 4.9. Cell-Lines 

Human cell-lines of hepatocellular carcinoma HepG-2 were cultivated in complete media under standard conditions in 5% CO_2_, 37 °C, and were free of mycoplasma [112]. DOX (Adriamycin)-resistant hepatic cell-lines HepG-2/ADR were developed by treating and maintaining the cells in media supplemented with 5 µg/mL DOX in the laboratories of King Abdullah University of Science and Technology, Thuwal at the Core Labs, Biological and Environmental Sciences and Engineering Division (BESE). Using RT-PCR, the development of DOX resistance through P-gp expression as compared to the parent sensitive cell-lines was confirmed. The DOX-free media were applied 7–10 days before conducting any experiments. 

### 4.10. Cytotoxicity and Reversal Assay

Exponentially growing cells (2 × 10^3^ cells/well) were seeded in 96-well plates for MTT cell viability assay [113]. The cells were grown for 24 h, then treated with serial concentrations of *Suaeda vermiculata* aq.-ethanolic extract (up 500 µg/mL) and DOX (100 µg/mL) for 24 h and with MTT solution (0.5 mg/mL) for 4 h. The DMSO dissolved the reaction product (crystals). Absorbance was determined at 570 nm using a SpectraMax M5e Multi-Mode Microplate Reader (Molecular Devices, LLC, USA). The same protocol was used to evaluate the cytotoxicity of the combination of DOX with *Suaeda vermiculata* aq.-ethanolic extract (20 µg/mL, a low, non-toxic dose). All experiments were carried out in triplicate. The IC_50_ values were determined. The Student’s *t*-test was applied to determine the degree of significant difference between the sets of data. The relative resistance (RR) for tested samples was calculated using the following equation:

(4) $$\mathrm{Relative}\;\mathrm{resistance}=\frac{{\mathrm{IC}}_{50\;}\mathrm{in}\;\mathrm{resistance}\;\mathrm{cells}}{{\mathrm{IC}}_{50\;}\mathrm{in}\;\mathrm{sensitive}\;\mathrm{cells}}$$

Combination index (CI): The nature of the interaction (synergistic, additive, or antagonistic) between extracts and DOX was determined by the combination index (CI).

(5) $$\mathrm{Combination}\;\mathrm{Index}=\frac{C_{\mathrm{DOX},50}}{{\mathrm{IC}}_{50,\;\mathrm{DOX}}}\;+\;\frac{C_{\mathrm{extract},50}}{{\mathrm{IC}}_{50,\mathrm{extract}}}$$

where C_DOX,50_ is the IC_50_ value for the cytotoxic agent in a two-drug combination, and C_extract_ is the fixed concentration of the extract, herein aq.-ethanolic extract. IC_50,DOX,_ and IC_50,extract_ corresponded to the IC_50_ for DOX, and aq.-ethanolic extract alone [114].

### 4.11. Statistical Analysis

Data were expressed as the mean ± standard error of the mean (SEM). Differences between groups were analyzed using two-way ANOVA followed by a posthoc test using Tukey’s multi-group comparison on GraphPad Prism 8.0.2, San Diego, U.S.A. The normality data were checked using GraphPad Prism. The data were considered significant if *p* < 0.05 [115]. The superscripts used to describe significance among the groups in the tables was obtained using Minitab 19.1. A Kolmogorov–Smirnov test was used for the determination of the normality of the data. 

## 5. Conclusions

The current study demonstrated the hepatoprotective potential and ameliorative effects of the *Suaeda vermiculata* aq.-ethanolic extract in CCl_4_-induced liver toxicity-bearing rat models. It also demonstrated the extract’s effective roles in cell line-based studies in controlling the damages from experimental liver cancer cell-lines. The aq.-ethanolic extract showed no adverse effects on the liver, kidney enzymes, glucose, or on the cholesterol levels in the experimental rats, though it showed a noticeable reducing effect on the triglycerides. The ongoing data on the concentrations of the antioxidant, and comparable liver-protective effects when equated with the silymarin and quercetin, confirmed the beneficial effects of the aq.-ethanolic extract in hepatoprotection, and also the safety of the extract at higher dose administrations. It also suggested that the consumption of the plant material by locals can be regarded as safe. The aq.-ethanolic extract has the potential to offer newer leads, and generate molecular templates upon intensive chemical investigation together with bioassay-guided biological activity pursuance towards finding a potential curative agent as a single molecular entity, or as part of the synergistic action of multiple molecules obtained from the plant’s extract for curing liver disorders at par, or higher levels of effectiveness in terms of reduced dose, and speed of recovery in comparison to the available silymarin derivatives, and other hepatoprotective synthetic products utilized for the purpose.

## Figures and Tables

**Table 1:** Identification of constituents from the aq.-ethanolic extract of *Suaeda vermiculata.*

Sr.	RT (min)	Mol. Formula	Calc. Mass	Obsrvd. Mass	Mass Fragments	Error (Δ ppm)	Compound Name	* R%	Refer
1.	16.11	C_10_H_16_O_5_	217.1076	217.10656 [M + H]	239.0883 [M^+^ + Na]; 455.1877 [2M + Na]	−2.265	Senecic acid	0.84	[41]
2.	20.24	C_28_H_30_O_16_	623.1612	623.1611 [M + H]^+^	446.9041[M-Pentose-acetyl];582.8751 [M-acetyl + 2H]	−1.007	Kaempferol-*O*- (acetyl) hexoside-pentoside	4.37	[42]
3.	21.66	C_15_H_10_O_7_	303.2443	303.0492 [M + H]^+^	289.1404 [M-CH_2_]	0	Quercetin	12.45	[43]
4.	23.45	C_15_H_8_O_6_	285.0399	285.04069 [M + H]^+^	284.99585 [M]; 286.04395 [M + 2H]	0.810	Rhein	1.26	[44]
5.	24.18	C_12_O_14_O_4_	223.0970	223.09634 [M + H]^+^	177.0543 [M + H-C_2_H_10_O]; 245.0782 [M + Na];467.16744 [2M + Na]	−0.957	4-Acetyl-6-(2-methylpropionyl)-1,3-resorcinol	2.32	[45]
6.	29.30	C_16_H_22_O_4_	279.1596	279.15900 [M + H]^+^	301.1407 [M + Na]; 579.2921 [2M + Na]	−0.323	(2E)-Heptyl-3-(3,4-dihydroxyphenyl) acrylate	3.37	[46]
7.	30.15	C_16_H_30_O_4_	287.2222	287.22140 [M + H]^+^	199.16898 [M-C_4_H_10_O_2_]; 309.20324 [M + Na]; 595.41675 [2M + Na]	−1.260	Hexadecanedioic acid	1.57	[47]
8.	31.70	C_55_H_74_N_4_O_5_	872.5815	872.1408 [M + H]^+^	256.26268 [Phytyl-C_2_H_5_ + H];280.26241 [phytyl moiety];593.15607 [M-phytyl];577.12459 [M-phytyl–methyl];615.13623 [M-phytyl + Na]	0.240	Pheophytin-a	23.69	[48]
9.	31.75	C_27_H_30_O_16_	633.1431	633.14559 [M + Na]^+^	634.14630 [M + H + Na]	4.719	Quercetin-3-O-rutinoside (Rutin)	7.80	[43]

* Relative % calculated based on the individual compound’s peak area and the chromatogram’s total peaks area.

**Table 2:** Total phenolics, total flavonoids of the extracts of *Suaeda vermiculata.*

Extracts	Extractive Values *	Total Phenolic Contents (mg GAE/g)	Total Flavonoid Contents (mg QE/g)
*n*-Hexane fraction	1.06	15.1 ± 0.60 ^E^	nd
Ethyl acetate fraction	4.37	317.5 ± 0.17 ^A^	13.80 ± 0.04 ^BC^
Chloroform fraction	1.27	140.0 ± 1.02 ^D^	17.47 ± 0.02 ^A^
*n*-Butanolic fraction	6.25	192.6 ± 0.97 ^C^	11.55 ± 0.02 ^C^
aq.-Ethanolic extract (mother liquor)	20.00	200.6 ± 2.14 ^B^	16.70 ±1.56 ^AB^

* Extractive values calculated as g/100 g of the dried plant powder; nd = not detected; GAE = gallic acid equivalent; QE = quercetin equivalent. The experiment was carried out in triplicates. Values were expressed as mean ± SEM. Means that do not share a letter are significantly different for the relevant column.

**Table 3:** Effect of aq.-ethanolic extract of *Suaeda vermiculata* on liver and kidney functions on carbon tetrachloride-induced liver toxicity in experimental rats.

Groups	AST IU/L	ALT IU/L	TP g/dL	Creatinine mg/dL
I. Intact control (no CMC, no drug, no aq.-ethanolic extract, CCl_4_-)	62.83 ±2.21 ^B^	67.42 ± 4.66 ^B^	7.39 ± 0.17 ^A^	0.91 ± 0.02 ^A^
II. aq.-Ethanolic extract, 250 mg/Kg, CCl_4_-	54.36 ± 3.64 ^B^	55.19 ± 3.47 ^B^	6.14 ± 0.34 ^BC^	0.80 ± 0.04 ^A^
III. Negative control (vehicle CMC 0.5%), CCl_4_+	169.57 ± 12.68 ^A^	103.38 ± 4.86 ^A^	7.23 ± 0.17 ^AB^	0.82 ± 0.03 ^A^
IV. Silymarin, 200 mg/kg, CCl_4_+	55.40 ± 2.45 ^B^	55.25 ± 2.91 ^B^	5.32 ± 0.14 ^BC^	0.82 ± 0.04 ^A^
V. aq.-Ethanolic extract, 100 mg/kg, CCl_4_+	65.35 ± 3.92 ^B^	71.77 ± 4.25 ^B^	7.64 ± 0.23 ^A^	0.80 ± 0.03 ^A^
VI. aq.-Ethanolic extract, 250 mg/kg, CCl_4_+	56.29 ± 3.30 ^B^	69.21 ± 3.79 ^B^	7.23 ± 0.27^AB^	0.86 ± 0.03 ^A^
VII. aq.-Ethanolic extract, 500 mg/kg, CCl_4_+	54.59 ± 1.67 ^B^	66.45 ± 7.05 ^B^	7.56 ± 0.32 ^A^	0.87 ± 0.02 ^A^
VIII. Quercetin, 100 mg/kg, CCl_4_+	49.54 ± 1.23 ^B^	52.39 ± 4.56 ^B^	8.05 ± 0.18 ^A^	0.82 ± 0.03 ^A^

Values denoted are mean ± SEM, *n* = 8 animals/group. AST: Aspartate transaminase, ALT: Alanine transaminase, TP: Total protein, CMC: Carboxyl methylcellulose. CCl_4−_: Carbon tetrachloride was not administered. CCl_4_+: Carbon tetrachloride was administered. Means that do not share a letter are significantly different for the relevant column.

**Table 4:** Effect of aq.-ethanolic extract of *Suaeda vermiculata* on blood glucose, triglycerides, and cholesterol on carbon tetrachloride (CCl_4_)-induced liver toxicity in experimental rats *.

Groups	Glucose mg/dL	Cholesterol mg/dL	Triglycerides mg/dL
I. Intact control (no CMC, no Drug, no aq.-ethanolic extract, CCl_4_-)	122.46 ± 2.66 ^A^	85.06 ± 1.85 ^A^	86.26 ± 2.24 ^BC^
II. aq.-Ethanolic extract, 250 mg/Kg, CCl_4_-	101.82 ± 4.47 ^AB^	78.73 ± 3.87 ^AB^	74.11 ± 2.95 ^C^
III. Negative control (vehicle CMC 0.5%), CCl_4_+	111.23 ± 3.23 ^AB^	61.70 ± 1.42 ^D^	100.20 ± 6.80 ^AB^
IV. Silymarin, 200 mg/kg, CCl_4_+	110.43 ± 6.43 ^AB^	65.94 ± 2.17 ^CD^	99.54 ± 4.28 ^AB^
V. aq.-Ethanolic extract, 100 mg/kg, CCl_4_+	99.86 ± 4.15 ^B^	59.46 ± 1.66 ^D^	76.56 ± 3.25 ^C^
VI. aq.-Ethanolic extract, 250 mg/kg, CCl_4_+	99.49 ± 7.00 ^B^	80.93 ± 2.08 ^AB^	97.53 ± 3.58 ^AB^
VII. aq.-Ethanolic extract, 500 mg/kg, CCl_4_+	98.79 ± 6.70 ^B^	66.66 ± 2.20 ^CD^	78.16 ± 2.62 ^C^
VIII. Quercetin 100 mg/kg, CCl_4_+	115.46 ± 3.58 ^AB^	72.83 ± 1.57 ^BC^	3.02 ^A^

* Values are denoted as mean ± SEM. CMC: Carboxyl methylcellulose. CCl_4_-: Carbon tetrachloride was not administered. CCl_4_+: Carbon tetrachloride was administered. Means that do not share a letter are significantly different for the relevant column.

**Table 5:** The IC_50_ values of *Suaeda vermiculata* aq.-ethanolic extract compared to DOX in HepG-2 wild types, and HepG-2/ADR resistant cell-lines, and their relative resistance (RR) using MTT [3-(4,5-dimethylthiazol-2-yl)-2,5-diphenyl tetrazolium bromide] assay.

Groups	IC_50_ (µg/mL) *		RR
	HepG-2	HepG-2/ADR	
aq.-Ethanolic extract	56.19 ± 2.55	78.40 ± 0.32 ***	1.39
Doxorubicin	1.3 ± 0.064	4.77 ± 1.05 ***	3.67

* Values expressed as mean ± SEM, *** = *p* < 0.001 using Student’s *t*-test.

**Table 6:** Synergistic interaction of a combination of DOX with 20 µg/mL of *Suaeda vermiculata* aq.-ethanolic extract in resistant HepG-2/ADR cell-lines *.

Groups	IC_50_ (µg/mL)	Synergistic Parameters			
	HepG-2/ADR	FR	CI	r	IB
Doxorubicin (DOX)	4.77 ± 1.05	--	--	--	--
DOX + aq.-ethanolic extract	1.72 ± 0.07 ***	2.77	0.56	0.99	Synergism

* Values were expressed as mean ± SEM. CI, combination index; FR, fold reversal; IB, isobologram; and medium effect equation (r-value). CI < 1 indicates synergism, CI = 1 indicates additive, and CI > 1 indicates antagonism. *** = *p* < 0.001 using Student’s *t*-test.
